# Understanding the role of the M1 muscarinic receptor in the propagation of tau as a pathology of Alzheimer's disease

**DOI:** 10.1002/alz70855_099012

**Published:** 2025-12-23

**Authors:** Katharine Gibson, Andrew B Tobin

**Affiliations:** ^1^ University of Glasgow, Glasgow, Please Select, United Kingdom; ^2^ University of Glasgow, Glasgow, United Kingdom

## Abstract

**Background:**

Neurofibrillary tangles (NFTs) composed of hyperphosphorylated tau are a hallmark of Alzheimer's disease (AD). While NFT propagation correlates with disease progression, the underlying molecular mechanisms are not well understood. Loss of cholinergic neurons and disrupted cholinergic signalling also occurs throughout AD. Previous studies show activation of the M1 muscarinic acetylcholine receptor (M1 mAChR) rescues learning and memory in neurodegeneration models. We aim to map M1 neuron and receptor expression to establish how M1 mAChR localisation relates to tau propagation patterns, and elucidate the role of this receptor in tau propagation.

**Method:**

To establish the neuro‐anatomy of M1+ neurons and receptor expression patterns, two genetically modified mouse models were used: (i) a Cre/loxP model with GFP expression under control of the M1 mAChR promoter indicating M1+ neurons, and (ii) a knock‐in model where the coding sequence for a M1 mAChR:eGFP fusion protein replaces the wild type M1 mAChR coding sequence enabling the receptor itself to be localised. Mice were perfused with 4% PFA, and 50µm brain slices were incubated with anti‐GFP primary antibody. For *in vitro* studies, 100,000 neurons were plated on PDL‐coated coverslips. 10µg human AD tau was seeded at DIV 7, and at DIV 14 neurons were fixed and immunostained for phospho‐tau antibodies. All images were acquired using an LSM880 confocal microscope.

**Result:**

High expression of the M1 mAChR and M1+ neurons occurs in the hippocampus, cortex, and amygdala. Expression also occurs throughout the lateral septum and cerebellum. There is differential expression in the hippocampus: M1+ neurons are seen in the pyramidal layer, while the receptor is at the membrane layer. Tau uptake and propagation was evident in M1‐WT neurons but was less extensive in M1‐KO controls.

**Conclusion:**

Regions important for learning and memory show high M1 expression, though locations of neuronal and receptor expression differ. Tau propagation was evident in M1‐WT neurons but limited in M1‐KO cultures, suggesting a role of the M1 mAChR in tau propagation. In future, hyperphosphorylated tau from human AD patients will be seeded *in vivo* in areas of high M1 expression via stereotactic injection.

